# Identity development and online and offline prosocial behaviors among early and middle adolescents

**DOI:** 10.3389/fpsyg.2023.1148347

**Published:** 2023-05-25

**Authors:** Yasuhiro Iwasa, Shogo Hihara, Kazuya Ishizaki, Genki Yasui, Makoto Hiro, Kazumi Sugimura

**Affiliations:** ^1^Division of Liberal Arts, Natural, Social, and Health Sciences, School of Science and Engineering, Tokyo Denki University, Saitama, Japan; ^2^Faculty of Business Administration, Matsuyama University, Matsuyama, Ehime, Japan; ^3^Graduate School of Humanities and Social Sciences, Hiroshima University, Hiroshima, Japan

**Keywords:** identity development, identity status, online prosocial behavior, offline prosocial behavior, variable-centered approach, person-centered approach, adolescence

## Abstract

Research has demonstrated that adolescents of the digital age engage in developmental tasks by interacting with others in both online and offline environments. However, no studies have investigated how adolescents develop their identity, a crucial developmental task, by engaging in online and offline prosocial behaviors. To address this research gap, we examined the role of online and offline prosocial behavior in identity development during adolescence using variable- and person-centered approaches. The participants were 608 individuals in early adolescence (50.2% girls; age range = 12–13 years, *M*_age_ = 12.75 years, *SD* = 0.43) and 594 individuals in middle adolescence (50.3% girls; age range = 15–16 years, *M*_age_ = 15.79 years, *SD* = 0.41) in Japan. They completed questionnaires to measure identity development, online and offline prosocial behavior, and demographic characteristics. The results from the variable-centered approach (i.e., identity dimensions) revealed that both online and offline prosocial behaviors were positively related to commitments and proactive explorations in early and middle adolescence. The findings from the person-centered approach (i.e., identity statuses) demonstrated that early and middle adolescents with higher levels of online prosocial behavior were more likely to show *searching moratorium* than all other identity statuses, whereas those with higher levels of offline prosocial behavior were more likely to show *achievement* than *troubled diffusion, carefree diffusion*, and *undifferentiated*. Consistent with both variable- and person-centered approaches, these findings highlight that online prosocial behavior can be a new resource for identity development in adolescence. Moreover, the results suggest that online prosocial behaviors lead to identity status in the process of maturing identity and that offline prosocial behavior is necessary to become more mature identity status. Regarding practical implications, educating adolescents on digital media literacy, including supportive behavior in online environments, is crucial to gradually exploring their identity. In addition, for adolescents to develop more mature identity, adults should create in-person environments in which they participate in offline prosocial behavior. The limitations of our findings with respect to the online and offline prosocial behavior scale items are discussed.

## 1. Introduction

Developing one’s identity in social contexts is a crucial developmental task in adolescence (Erikson, 1968; Branje et al., 2021). Previous studies have revealed that identity development is prompted by offline prosocial behavior in in-person environments, which involves social responsibility and agency (e.g., Hardy et al., 2011). In today’s increasingly internet-based technological society, however, adolescents engage in developmental tasks through online as well as offline social interactions (Ehrenreich et al., 2021). Despite this, whether adolescents develop identity through online prosocial behavior as they develop it through offline prosocial behavior has not yet been explored. To address this research gap, we investigated the role of both online and offline prosocial behaviors in identity development among adolescents. Providing novel evidence on this issue can uncover a resource for adolescents’ adaptive development in the digital age.

### 1.1. Identity development

Theory of lifespan development of Erikson (1968) assumes that adolescents develop their identity by resolving the conflict between synthesis (i.e., a coherent sense of self) and confusion (i.e., a fragmented and changeable sense of self). Based on Erikson’s theory, Marcia (1966) posits two key components of identity development: *exploration* and *commitment*. Exploration refers to the search for goals, beliefs, and values in life, whereas commitment refers to making firm choices about and engaging in these goals, beliefs, and values. Contemporary identity development research has proposed models that extend Marcia’s model (see Crocetti and Meeus, 2015, for a review). We adopt a five-dimensional model (Luyckx et al., 2008), which can capture more detailed processes of identity development in adolescence. This model consists of three exploration processes and two commitment processes in the domain of future plans, which is one of the most important issues for adolescents (Nurmi, 1991; Hihara et al., 2021). *Exploration in breadth* represents actively seeking various future plans as identity options. *Commitment making* refers to making a few choices regarding future plans as identity options. *Exploration in depth* represents the careful evaluation of selected future plans. *Identification with commitment* refers to feeling certain about the selected future plans. *Ruminative exploration* represents continuing to worry about future plans. Previous studies have revealed relationships between identity dimensions and various aspects of adaptation (e.g., Luyckx et al., 2008; Ritchie et al., 2013; Hatano et al., 2020). Commitment making and identification with commitment (i.e., commitments) were positively associated with adaptation (e.g., subjective well-being and self-esteem) and negatively associated with maladaptation (e.g., depression and delinquency). Exploration in breadth and exploration in depth were positively associated with adaptation, while ruminative exploration was negatively associated with adaptation and positively associated with maladaptation. Exploration in breadth and exploration in depth are recognized as proactive explorations to distinguish them from rumination exploration as a maladaptive exploration (Bogaerts et al., 2019).

This model can also be used to classify individuals into different identity statuses based on combinations of the five identity dimensions (Luyckx et al., 2008; Sugimura, 2020; see Table 1). Identity statuses represent maturation stages according to individual differences in addressing identity-related issues (Meeus et al., 2010). Individuals in the *achievement* status have high levels of commitments and proactive explorations, and low level of ruminative exploration. Those in the *foreclosure* status exhibit moderate to high levels of commitments and low levels of proactive and ruminative explorations. Individuals in the *moratorium* status show low to moderate levels of commitments and high levels of proactive and ruminative explorations. Individuals in the *searching moratorium* status show high levels of all five identity dimensions. Those in the *troubled diffusion* status possess low levels of commitments and proactive explorations, and high level of ruminative exploration. Individuals in the *carefree diffusion* status have low levels of all five dimensions. Individuals in the *undifferentiated* status have moderate levels of all five dimensions. *Achievement* and *troubled diffusion* and *carefree diffusions* represent the most mature identity status and the least mature identity statuses endpoints of the developmental continuum, respectively, and *moratorium*, *searching moratorium*, *foreclosure*, and *undifferentiated* represent statuses in intermediate developmental stages (Luyckx et al., 2008; Meeus et al., 2010). Concerning identity statuses and various indicators of adaptation, previous studies showed that *achievement* and *foreclosure*, which were high commitments, were the most adaptive statuses (e.g., high life satisfaction and low depression), *moratorium, searching moratorium*, and *undifferentiated* were the next most adaptive statuses, and *troubled diffusion and carefree diffusion* were the least adaptive statuses (Luyckx et al., 2008; Schwartz et al., 2011; Hatano and Sugimura, 2017). In identity development research, it is important to acquire a comprehensive understanding of identity development using both variable- and person-centered approaches (Crocetti and Meeus, 2015). A variable-centered approach can identify the factors involved in each identity dimension during early and middle adolescence. A person-centered approach can reveal factors that lead to characteristic profiles in identity development (i.e., identity statuses) among early and middle adolescents. Variable- and person-centered approaches differ in the aspects they uncover and can complement each other. Therefore, we examined the relationship between identity development and prosocial behavior using both variable- and person-centered approaches.

**Table 1:** Identity statuses in the five identity dimensions model.

	Achievement	Foreclosure	Moratorium	Searching moratorium	Troubled diffusion	Carefree diffusion	Undifferentiated
Commitment making	High	Moderate to high	Low to moderate	High	Low	Low	Moderate
Identification with commitment	High	Moderate to high	Low to moderate	High	Low	Low	Moderate
Exploration in breadth	High	Low	High	High	Low	Low	Moderate
Exploration in depth	High	Low	High	High	Low	Low	Moderate
Ruminative exploration	Low	Low	High	High	High	Low	Moderate

Patterns of identity statuses were summarized from Luyckx et al. (2008), Nakama et al. (2015), and Sugimura (2020).

### 1.2. Online and offline prosocial behaviors

Prosocial behavior is a voluntary action intended to benefit others (Eisenberg et al., 2006), such as helping, comforting, and rescuing others. With the development of perspective taking in adolescence, adolescents have more opportunities for prosocial behavior by showing empathic concern for others’ feelings (Van der Graaff et al., 2018). Prior research has shown that prosocial behavior in adolescence predicts positive developmental outcomes, such as self-esteem (Fu et al., 2017). With the establishment of various online platforms, recent studies have examined prosocial behavior separately in offline and online contexts (Erreygers et al., 2018; Armstrong-Carter and Telzer, 2021). Offline prosocial behavior refers to prosocial behavior that occurs in offline situations where people meet face-to-face. Online prosocial behavior refers to prosocial behavior conducted using online platforms such as social networking services. In today’s growing internet environment, adolescents have more opportunities to engage in prosocial behaviors on online platforms, where there are fewer social and temporal barriers, than offline prosocial behaviors made in in-person environments (Armstrong-Carter and Telzer, 2021). Therefore, through online prosocial behavior, adolescents form social connections with others in online environments that they cannot obtain in the offline environments. These online connections may help adolescents address various developmental issues (Moreno and Uhls, 2019).

### 1.3. The role of online and offline prosocial behaviors in identity development among early and middle adolescents

Considering previous studies, identity development in adolescence may be promoted by both online and offline prosocial behaviors. Adolescents’ offline prosocial behavior involves social responsibility and agency (Yates and Youniss, 1996). Accordingly, previous research has demonstrated that offline prosocial behavior promotes proactive explorations and commitments, which are related to matured identity status (i.e., *achievement*; Pancer et al., 2007; Hardy et al., 2011). Based on co-construction model (Subrahmanyam et al., 2006) indicating that adolescents engage in developmental tasks through online social interactions as they do offline, online prosocial behavior is expected to promote identity development as does offline prosocial behavior. Moreover, considering the affordance approach, which suggests that online environments may offer unique opportunities for adolescent development (Moreno and Uhls, 2019), it is possible that online prosocial behavior in adolescence may relate to identity development in a distinct way compared to offline prosocial behavior. The age of adolescents who engage in online prosocial behavior may also need to be considered. This is because early adolescents, who impulsively and sensitively seek novel experiences, may not use the internet effectively (Liu et al., 2022). Online prosocial behavior in early adolescence has been reported to be associated with online antisocial behavior (Erreygers et al., 2018), suggesting that early adolescents tend to lack responsibility and positive goals in society. Therefore, in early adolescence, online prosocial behavior may lead to ruminative exploration as well as proactive explorations and commitments, and may be related to identity status in the process of maturing identity (i.e., *searching moratorium*). Despite these assumptions, there is no empirical research on the role of both online and offline prosocial behaviors in identity development, considering developmental stages in adolescence.

### 1.4. The present study

This study aimed to comprehensively investigate the role of online and offline prosocial behaviors in identity development among early and middle adolescents. First, using a variable-centered approach, we examined the relationship between online and offline prosocial behaviors and five identity dimensions. We hypothesized that in both early and middle adolescence, online and offline prosocial behaviors would be positively related to commitments and proactive explorations in the identity dimensions (Hypothesis 1a). We also hypothesized that online prosocial behavior would be positively related to ruminative explorations in the identity dimensions only in early adolescence (Hypothesis 1b). Second, using a person-centered approach, we examined the relationship between online and offline prosocial behaviors and identity statuses. We hypothesized that early adolescents with higher levels of online prosocial behavior would be more likely to show an identity status with high commitments and proactive and ruminative explorations (i.e., *searching moratorium*) than other statuses (Hypothesis 2a). Meanwhile, middle adolescents with higher levels of online prosocial behavior would be more likely to show an identity status with high commitments and proactive explorations, and low ruminative exploration (i.e., *achievement*) than other statuses (Hypothesis 2b). We also hypothesized that both early and middle adolescents with higher levels of offline prosocial behavior would be more likely to show *achievement* than other statuses (Hypothesis 2c).

## 2. Materials and methods

### 2.1. Participants

The participants were 608 early adolescents (first-grade junior high school students; age range 12–13 years, *M*_age_ = 12.8, *SD* = 0.4) and 594 middle adolescents^1^ (first-grade high school students; age range 15–16 years, *M*_age_ = 15.8, *SD* = 0.4) in Japan. Table 2 presents the demographic characteristics. Among early adolescents, 49.8% were men and 50.2% were women, and among middle adolescents, 49.7% were men and 50.3% were women. The sample was diverse in terms of family income, parents’ educational level, and geographic region. Regarding annual family income, families with 4–6 million JPY (29,630–44,444 USD) were the most common among early adolescents, while families with 6–8 million JPY (44,444–59,259 USD) were the most common among middle adolescents. Regarding parents’ educational level, 69.9% of fathers and 74.5% of mothers among early adolescents had completed higher education (i.e., university, graduate school, technical school, and junior college), whereas 69.4% of fathers and 74.9% of mothers among middle adolescents had completed higher education. As for geographic region, most early (71.2%) and middle (75.1%) adolescents lived in relatively urban areas (i.e., Kanto, Chubu, and Kinki districts) in Japan.

**Table 2:** Demographic information of the present sample.

Demographic characteristics	Detail	Number (%)	
		Early adolescents (*n* = 608)	Middle adolescents (*n* = 594)
Age	12	153 (25.2)	-
	13	455 (74.8)	-
	15	-	127 (21.4)
	16	-	467 (78.6)
Sex	Men	303 (49.8)	295 (49.7)
	Women	305 (50.2)	299 (50.3)
Family income	Less than 2 million JPY (14,815 USD)	9 (2.0)	8 (1.8)
	2–4 million JPY (14,815–29,630 USD)	68 (14.8)	55 (12.7)
	4–6 million JPY (29,630–44,444 USD)	155 (33.7)	102 (23.6)
	6–8 million JPY (44,444–59,259 USD)	105 (22.8)	105 (24.2)
	8–10 million JPY (59,259–74,074 USD)	59 (12.8)	78 (18.0)
	10–12 million JPY (74,704–88,889 USD)	28 (6.1)	38 (8.8)
	12–15 million JPY (88,889–111,111 USD)	18 (3.9)	25 (5.8)
	15–20 million JPY (111,111–148,148 USD)	11 (2.4)	14 (3.2)
	More than 20 million JPY (148,148 USD)	7 (1.5)	8 (1.8)
	Missing	148 (24.3)	161 (27.1)
Father’s educational level	Secondary education	175 (28.8)	171 (28.8)
	Higher education	425 (69.9)	412 (69.4)
	Missing	8 (1.3)	11 (1.8)
Mother’s educational level	Secondary education	153 (25.2)	146 (24.6)
	Higher education	453 (74.5)	445 (74.9)
	Missing	2 (0.3)	3 (0.5)
Region	Urban areas	433 (71.2)	446 (75.1)
	Rural areas	175 (28.8)	148 (24.9)

### 2.2. Procedure

We collected data using a survey research company (MACROMILL; https://www.macromill.com/), which works with a variety of registrants globally (e.g., the United States, Europe, and Asia). At the beginning of the online survey, the company sent an email to registrants that matched the researchers’ request. For this survey, we made the following requests to the company: (a) Japanese nationals, (b) from diverse regions of Japan, and (c) approximately 600 individuals in both early and middle adolescent groups. Because adolescents younger than 18-year-old could not register for the research company themselves, an email was sent to parents with a child in the first grade of junior high school or high school. Registrants (i.e., parents) matching these researchers’ requests received an e-mail that included (a) the study’s purpose and (b) a hyperlink to the online survey. After both adolescents and parents signed an informed consent agreement, they answered the online survey. This study was approved by the Ethical Review Board of Hiroshima University in Japan.

### 2.3. Measures

#### 2.3.1. Identity development

Identity development was assessed using the Dimensions of Identity Development Scale (DIDS; Luyckx et al., 2008; for the Japanese version, see Nakama et al., 2015). This scale comprises 25 items assessing the five dimensions of identity development, with five items for each dimension: commitment making (e.g., “I have decided on the direction I want to follow in my life”), identification with commitment (e.g., “My future plans give me self-confidence”), exploration in breadth (e.g., “I think willingly about what kind of life I am going to lead”), exploration in depth (e.g., “I work out for myself if the goals I put forward in life really suit me”), and ruminative exploration (e.g., “I keep looking for the direction I want to take in my life”). All items are rated on a five-point Likert-type scale ranging from 1 (completely untrue) to 5 (completely true). We used average scores. In the early adolescent sample, the Cronbach’s alphas were 0.91 for commitment making, 0.86 for identification with commitment, 0.87 for exploration in breadth 0.84 for exploration in depth, and 0.79 for ruminative exploration. In the middle adolescent sample, the Cronbach’s alphas were 0.92 for commitment making, 0.89 for identification with commitment, 0.88 for exploration in breadth 0.81 for exploration in depth, and 0.84 for ruminative exploration.

#### 2.3.2. Online prosocial behavior

We used the “performing online prosocial behavior” subscale of the Online Prosocial Behavior Scale (OPBS; Erreygers et al., 2018). This scale captures behavioral dispositions rather than behaviors themselves. The Japanese version of OPBS was developed using a back-translation method. Specifically, the second author and a research assistant first translated the measure from English to Japanese. One bilingual professional translator then back-translated the Japanese version into English. Another bilingual professional translator carefully compared the original with the back-translated items and confirmed that they were consistent. In each of these steps, differences in translation were discussed by the first, second, and third authors and disagreements were resolved through discussion. This subscale comprises 10 items (e.g., “Say nice/friendly things to someone”), which are scored using a five-point Likert-type scale, ranging from 1 (never) to 5 (every day). As the instruction, we asked, “How often have you [done]/[experienced] the following via electronic media (smartphone, computer, tablet…) in the past month?” We used average scores. The Cronbach’s alphas were 0.91 and 0.90 for the early and middle adolescents, respectively.

#### 2.3.3. Offline prosocial behavior

We used the prosocial behavior subscale of the self-rated Strength and Difficulty Questionnaire (SDQ; Goodman, 1997, for the Japanese version, see Youth in mind, 2022). This subscale captures behavioral tendencies rather than behaviors themselves. This subscale comprises five items (e.g., “I am helpful if someone is hurt, upset, or feeling ill”), which are scored using a three-point Likert-type scale, ranging from 0 (not true) to 2 (certainly true). The instruction was “Please think about your last 6 months or so and answer the question.” We used average scores. The Cronbach’s alphas among early and middle adolescents were 0.63 and 0.73, respectively.

#### 2.3.4. Internet usage time

Regarding internet usage time, the questionnaires included three items developed based on measures in previous studies (Wright and Li, 2011; Cabinet Office in Japan, 2022). We asked participants their average daily time in the past month spent on (1) social media (LINE, Twitter, Instagram, Facebook, TikTok, etc.), (2) online communications through phone calls, emails, and text messages (excluding LINE), and (3) overall internet usage time, including time spent on (1) and (2). Participants rated the items on a scale of 1–8 (1 = less than 1 h, 8 = more than 7 h). In this study, we used only the item assessing (3) overall internet usage time as an indicator of internet usage time. Table 3 shows the distribution of internet usage time. The largest proportion of early and middle adolescents answered less than 1 h (37.2%) and 2–3 h (24.1%), respectively.

**Table 3:** Distributions of internet usage time.

Internet usage time	Number (%)	
	Early adolescents (*n* = 608)	Middle adolescents (*n* = 594)
Less than 1 h	226 (37.2)	132 (22.2)
Less than 1–2 h	147 (24.2)	137 (23.1)
Less than 2–3 h	112 (18.4)	143 (24.1)
Less than 3–4 h	79 (13.0)	72 (12.1)
Less than 4–5 h	18 (3.0)	53 (8.9)
Less than 5–6 h	12 (2.0)	25 (4.2)
Less than 6–7 h	8 (1.3)	10 (1.7)
More than 7 h	6 (1.0)	22 (3.7)

### 2.4. Statistical analyses

Statistical analyses were conducted using SPSS version 24 (Arbuckle, 2016) and Mplus version 8.7 (Muthén and Muthén, 2021). As we examined the relationship between identity development and online and offline prosocial behaviors in two age groups, we tested measurement invariance for these variables between the two age groups. Three levels of invariance were tested: configural invariance (factor structures are equivalent across age groups), metric invariance (factor loadings are equivalent across age groups), and scalar invariance (factor loadings and item intercepts are equivalent across age groups). We evaluated the model fit using the comparative fit index (CFI) and the root mean square error of approximation (RMSEA). CFI values ≥0.900 and RMSEA values ≤0.080 indicate an acceptable fit (Byrne, 2012). To compare the models, we considered the Satorra-Bentler scaled chi-square (χ2 SB) difference test (Satorra and Bentler, 2001) and changes in CFI (ΔCFI) and RMSEA (ΔRMSEA; Cheung and Rensvold, 2002). To establish invariance between models, at least two of the following three criteria had to be met: non-significant Δχ2 SB (Satorra and Bentler, 2001), ΔCFI ≥ −0.010, and ΔRMSEA ≤ 0.015 (Chen, 2007). We used a maximum likelihood robust estimator (Satorra and Bentler, 2001) to deal with slight deviations from a normal distribution for the study variables.

**Figure 1:**
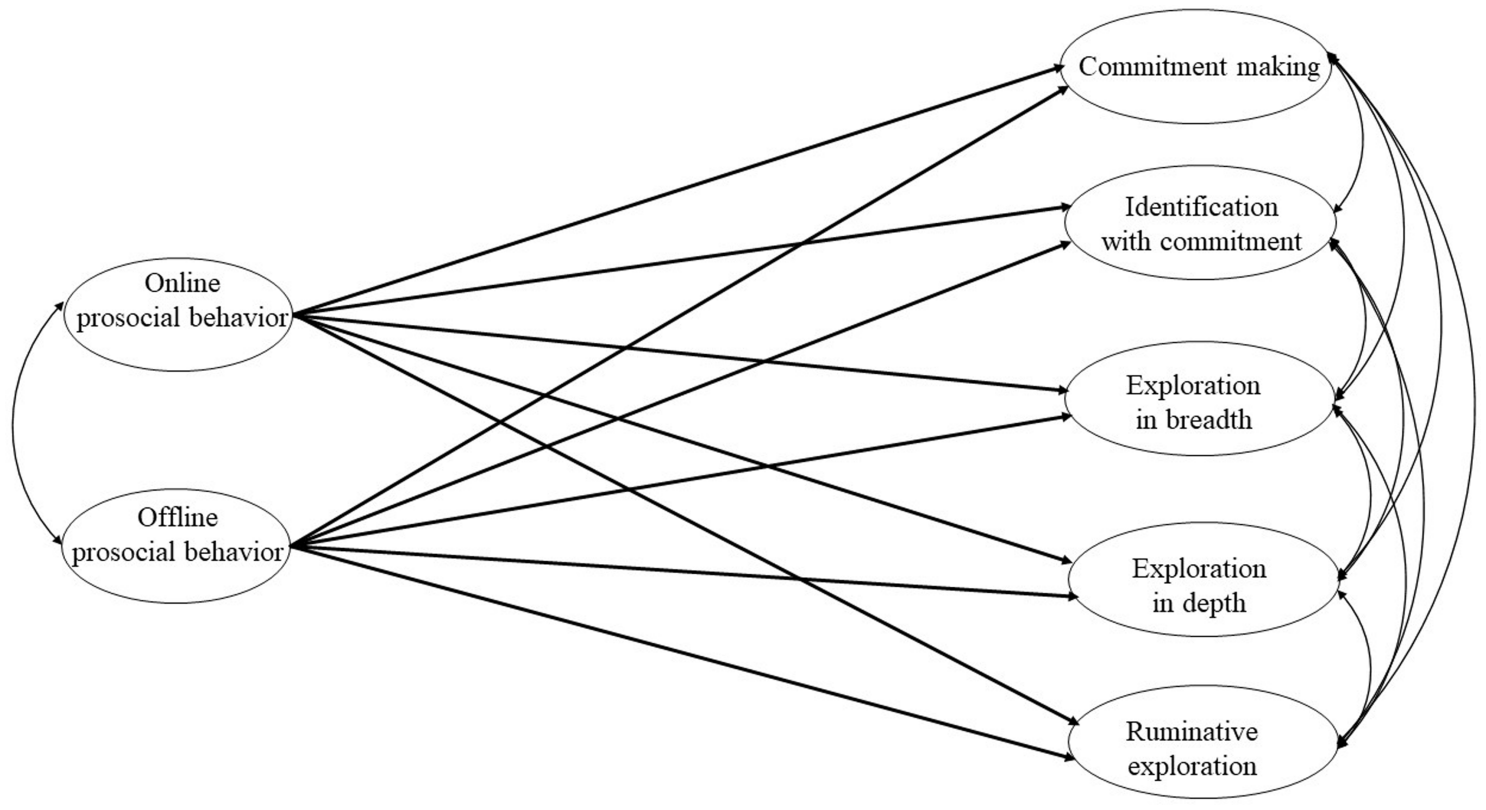
Path analyses testing the relationships between online and offline prosocial behavior and identity development. For the sake of clarity, paths linking control variables (i.e., internet usage time, sex, family income, father’s and mother’s education, and region) to study variables (i.e., identity development, online prosocial behavior, and offline prosocial behavior) are not displayed. ^*^p < 0.05, ^**^p < 0.01, and ^***^p < 0.001.

We conducted path analyses to examine the role of online and offline prosocial behaviors in the identity dimensions in a variable-centered approach. The model included online and offline prosocial behaviors as independent variables and five identity dimensions as dependent variables. Internet usage time, sex (0 = men; 1 = women), family income, father’s education (0 = secondary education; 1 = higher education), mother’s education (0 = secondary education; 1 = higher education), and region (0 = relatively urban areas; 1 = relatively rural areas) were included as control variables. To test whether the path coefficients differed between age groups, we conducted a multi-group analysis. The constrained model, in which all path coefficients were fixed to be equal between age groups was compared with the unconstrained model, in which these paths could vary across groups. To compare these models, we evaluated the model fit using ΔCFI, ΔRMSEA, and Δχ2 SB. If at least two of the three criteria (i.e., non-significant Δχ2 SB, ΔCFI ≥ −0.010, and ΔRMSEA ≤ 0.015) were met, the constrained model was adopted (Figure 1).

To examine the role of online and offline prosocial behavior in identity statuses using a person-centered approach, we classified all participants into identity statuses using cluster analyses with a two-step procedure (Gore, 2000). In the first step, hierarchical cluster analyses using Ward’s method based on squared Euclidean distance were performed to identify the optimal number of clusters. We compared 2–8-cluster solutions and selected the number of clusters based on three criteria: (1) theoretical meaningfulness of each cluster, (2) parsimony, and (3) explanatory power (i.e., the cluster solution had to explain approximately 50% of the variance in the identity dimensions). In the second step, an iterative *k*-means clustering method was performed using these initial cluster centers as non-random starting points. To test the stability of this cluster solution, we conducted a double-split cross-validation procedure (Breckenridge, 2000). We randomly split the entire sample into two subsamples and repeated the two-step cluster analyses. We used kappa (κ) coefficients to examine whether the clusters in these subsamples were consistent with those of the entire sample. To test the associations of online and offline prosocial behaviors with identity statuses, we performed a multinomial logistic regression analysis. The independent variables were online and offline prosocial behaviors, and the dependent variables were identity statuses. Internet usage time, sex (0 = men; 1 = women), family income, father’s education (0 = secondary education; 1 = higher education), mother’s education (0 = secondary education; 1 = higher education), and region (0 = relatively urban areas; 1 = relatively rural areas) were included as control variables. We also conducted a multi-group analysis to test whether the path coefficients differed between the age groups. The constrained model, in which all path coefficients were fixed to be equal between age groups was compared with the unconstrained model, in which these path coefficients could vary across groups. To compare each model, we evaluated the model fit by using Δχ2 SB. If the model comparison showed a non-significant Δχ2 SB, the constrained model was adopted.

## 3. Results

### 3.1. Preliminary analyses

The results of the measurement invariance test across age groups established scalar invariance for identity development, online prosocial behavior, and offline prosocial behavior (see Supplementary Table S1 in the Supplementary material).

Table 4 and Supplementary Table S2 present the descriptive statistics of the study variables. Middle adolescents scored significantly higher on commitment making, identification with commitment, exploration in breadth, exploration in depth, and online prosocial behavior than did early adolescents. Early adolescents scored significantly higher on offline prosocial behavior than did middle adolescents. Table 5 shows the correlations among the study variables. All five dimensions of identity development were positively correlated with online prosocial behavior (*r* = 0.13–0.37) and offline prosocial behavior (*r* = 0.07–0.32).

**Table 4:** Descriptive statistics for the study variables.

Variables	Total	Age differences			
		Early adolescents	Middle adolescents		
	*M* (*SD*)	*M* (*SD*)	*M* (*SD*)	*t*-values	Cohen’s *d*
Commitment making	3.03 (1.03)	2.91 (1.02)	3.14 (1.02)	*t* (1200) = 3.87^***^	0.22
Identification with commitment	3.10 (0.90)	3.04 (0.89)	3.16 (0.91)	*t* (1200) = 2.28^**^	0.13
Exploration in breadth	3.24 (0.89)	3.20 (0.89)	3.28 (0.89)	*t* (1200) = 1.60	0.09
Exploration in depth	3.04 (0.87)	2.98 (0.88)	3.11 (0.86)	*t* (1200) = 2.67^**^	0.15
Ruminative exploration	3.15 (0.80)	3.11 (0.76)	3.19 (0.84)	*t* (1200) = 1.79	0.10
Online prosocial behavior	2.63 (1.00)	2.55 (1.02)	2.71 (0.96)	*t* (1200) = 2.92^**^	0.17
Offline prosocial behavior	1.07 (0.45)	1.09 (0.43)	1.04 (0.47)	*t* (1200) = 2.06^*^	0.12

M, mean; SD, standard deviation.

^*^p < 0.05, ^**^p < 0.01, ^***^p < 0.001.

**Table 5:** Correlations between the study variables.

Variables		1	2	3	4	5	6	7	8
1. Commitment making		–							
2. Identification with commitment	*r*	0.84^***^							
	95% CI	[0.82, 0.86]							
3. Exploration in breadth	*r*	0.65^***^	0.70^***^						
	95% CI	[0.62, 0.68]	[0.67, 0.73]						
4. Exploration in depth	*r*	0.72^***^	0.75^***^	0.77^***^					
	95% CI	[0.69, 0.74]	[0.72, 0.77]	[0.75, 0.79]					
5. Ruminative exploration	*r*	−0.05	−0.02	0.25^***^	0.23^***^				
	95% CI	[−0.10, 0.01]	[−0.07, 0.04]	[0.19, 0.30]	[0.18, 0.28]				
6. Online prosocial behavior	*r*	0.31^***^	0.31^***^	0.33^***^	0.37^***^	0.13^***^			
	95% CI	[0.25, 0.36]	[0.26, 0.36]	[0.28, 0.38]	[0.32, 0.42]	[0.08, 0.19]			
7. Offline prosocial behavior	*r*	0.25^***^	0.29^***^	0.32^***^	0.32^***^	0.07^*^	0.36^***^		
	95% CI	[0.20, 0.30]	[0.24, 0.34]	[0.26, 0.37]	[0.26, 0.37]	[0.01, 0.13]	[0.31, 0.40]		
8. Internet usage time	ρ	−0.04	−0.05	0.01	−0.03	0.07^*^	0.13^***^	−0.01	
	95% CI	[−0.10, 0.02]	[−0.11, 0.00]	[−0.05, 0.06]	[−0.08, 0.03]	[0.01, 0.13]	[0.07, 0.18]	[−0.07, 0.05]	
9. Family income	ρ	0.12^***^	0.12^***^	0.13^***^	0.14^***^	−0.03	0.16^***^	0.07^*^	0.00
	95% CI	[0.06, 0.19]	[0.06, 0.19]	[0.07, 0.20]	[0.07, 0.20]	[−0.09, 0.04]	[0.09, 0.22]	[0.01, 0.14]	[−0.07, 0.06]

*r*, Pearson product–moment correlation coefficient; ρ, Spearman’s rank correlation coefficient; 95% CI, 95% confidence.

^*^p < 0.05, ^**^p < 0.01, and ^***^p < 0.001.

### 3.2. A variable-centered approach: the role of online and offline prosocial behaviors in identity dimensions

Path analyses were used to examine the role of online and offline prosocial behavior in identity dimensions. The model fit was good (χ2 SB = 790.866, *df* = 350, *p* < 0.001, CFI = 0.964, RMSEA = 0.038, 90%CI [0.034–0.041]). As reported in Table 6, both online and offline prosocial behaviors were positively related to commitment making, identification with commitment, exploration in breadth, and exploration in depth. Online and offline prosocial behaviors were not associated with ruminative exploration. We also conducted a multi-group analysis to test whether the paths differed between age groups. Model comparisons between the constrained and unconstrained models showed no significant differences between models (Δχ2 SB = 83.45, *df* = 52, *p* = 0.003, ΔCFI = −0.003, and ΔRMSEA = −0.001), suggesting that age differences between early and middle adolescence did not moderate the relationship between online and offline prosocial behaviors and identity dimensions.

**Table 6:** Standardized path coefficients for path analyses.

Independent variables	Estimates	Dependent variables				
		Commitment making	Identification with commitment	Exploration in breadth	Exploration in depth	Ruminative exploration
Main variables						
Online prosocial behavior	β	0.26^***^	0.25^***^	0.21^***^	0.27^***^	0.09
	95% CI	[0.15, 0.37]	[0.14, 0.37]	[0.10, 0.32]	[0.16, 0.38]	[−0.03, 0.21]
Offline prosocial behavior	β	0.20^***^	0.28^***^	0.33^***^	0.29^***^	0.04
	95% CI	[0.08, 0.33]	[0.15, 0.40]	[0.21, 0.45]	[0.17, 0.42]	[−0.10, 0.17]
Control variables						
Internet usage time	β	–	–	–	–	0.02
	95% CI					[−0.07, 0.11]
Sex (0 = men; 1 = women)	β	0.04	–	0.06^*^	0.06^**^	0.05
	95% CI	[−0.01, 0.09]		[−0.01, 0.09]	[0.03, 0.12]	[−0.04, 0.15]
Family income	β	0.03	0.02	0.04	0.05	–
	95% CI	[−0.07, 0.12]	[−0.07, 0.11]	[−0.05, 0.13]	[−0.04, 0.13]	
Father’s education level (0 = secondary school; 1 = higher school)	β	0.04	0.03	0.03	0.03	–
	95% CI	[−0.05, 0.14]	[−0.06, 0.12]	[−0.06, 0.13]	[−0.06, 0.12]	
Mother’s education level (0 = secondary education; 1 = higher education)	β	0.05	0.04	0.04	0.02	–
	95% CI	[−0.05, 0.14]	[−0.06, 0.13]	[−0.05, 0.14]	[−0.07, 0.12]	
Adjusted *R*^2^		0.18^***^	0.22^***^	0.24^***^	0.27^***^	0.02

β, standardized coefficient; 95% CI, 95% confidence; The paths from control variables to dependent variables were predicted only for those that were found to be associated in the preliminary analyses.

^*^p < 0.05, ^**^p < 0.01, and ^***^p < 0.001.

### 3.3. A person-centered approach: the role of online and offline prosocial behaviors in identity statuses

**Figure 2:**
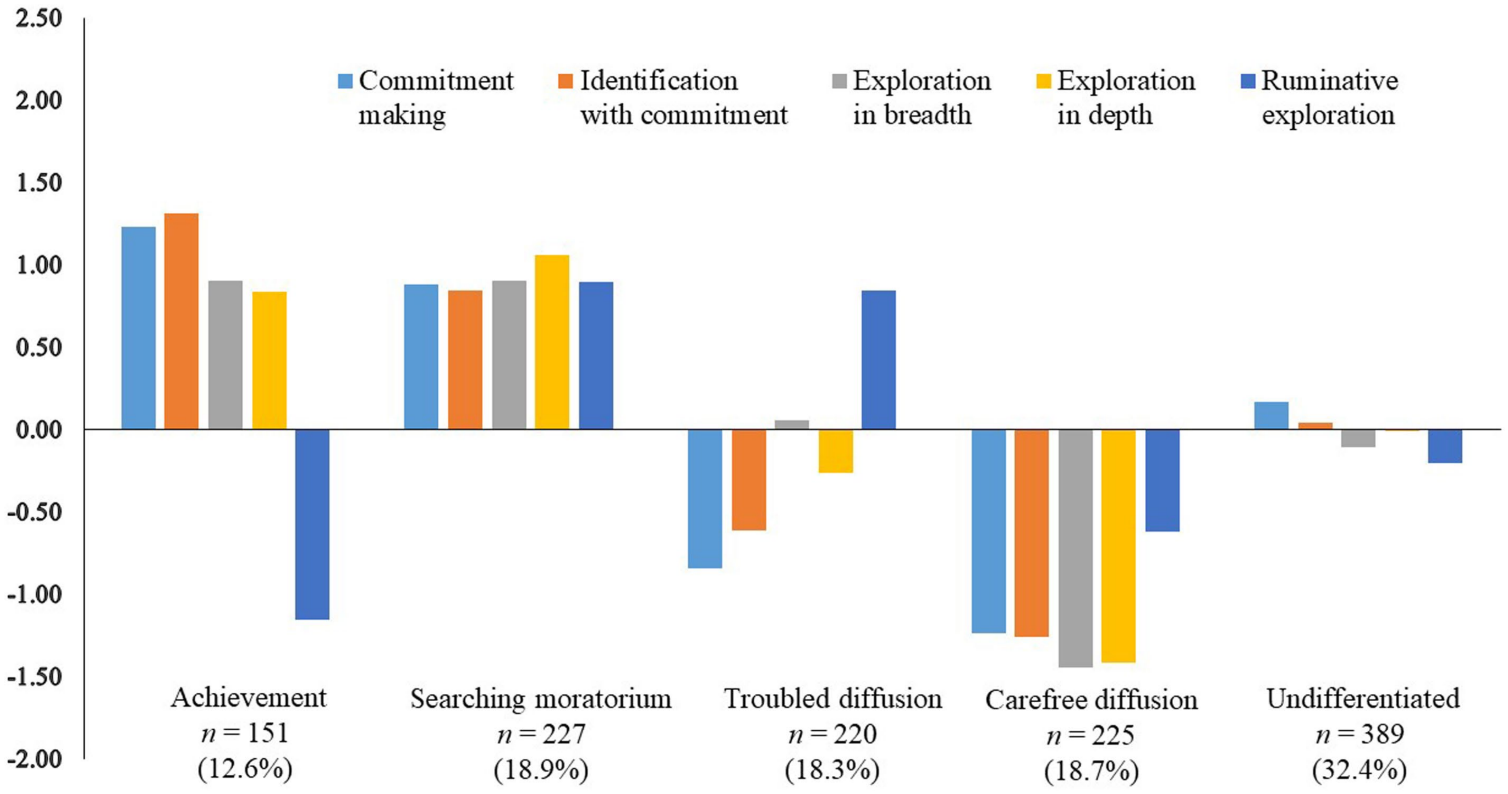
Z-scores for dimensions of identity development for the final five-cluster solutions.

Two-step cluster analysis indicated that the five-cluster solution was the most acceptable (Figure 2). These clusters showed the theoretically meaningful identity statuses found in previous studies, as follows. *Achievement* was adolescents with high commitments and proactive explorations. *Searching moratorium* was adolescents with high commitments and proactive and ruminative explorations. *Troubled diffusion* was adolescents with low commitments and proactive explorations, and high ruminative exploration. *Carefree diffusion* was adolescents with low commitments and proactive and ruminative explorations. *Undifferentiated* was adolescents with intermediate commitments and proactive and ruminative explorations. Regarding parsimony, one cluster in the six-cluster solution was similar to *undifferentiated* and did not add variation. The five-cluster solution explained 75, 71, 63, 67, and 53% of the variance in commitment making, identification with commitment, exploration in breadth, exploration in depth, and ruminative exploration, respectively. Furthermore, a double-split cross-validation procedure extracted the same five-cluster solution in both subsamples. The cluster assignment for the entire sample was highly consistent with that in each of the two subsamples (κs = 0.97 and 0.86).

Table 7 shows the odds ratios (ORs) for the associations of online and offline prosocial behaviors with identity statuses. Adolescents with higher levels of online prosocial behavior were more likely to show *searching moratorium* than *achievement*, *undifferentiated*, *troubled diffusion*, and *carefree diffusion*. They were also more likely to show *achievement* than *undifferentiated*, *troubled diffusion*, and *carefree diffusion*, and to show *undifferentiated* than *troubled diffusion* and *carefree diffusion*. Adolescents with higher levels of offline prosocial behavior were more likely to show *achievement* than *troubled diffusion*, *undifferentiated*, and *carefree diffusion*. They were also more likely to show *searching moratorium* than *undifferentiated* and *carefree diffusion*. Additionally, they were more likely to show *troubled diffusion* than *undifferentiated* and *carefree diffusion*. We also conducted a multi-group analysis to test whether the ORs differed between the age groups. Model comparison between the constrained and unconstrained models showed no differences between models (Δχ2 SB = 28.49, *df* = 32, *p* = 0.862), suggesting that age differences between early and middle adolescence did not moderate the relationship between online and offline prosocial behaviors and identity statuses.

**Table 7:** Analyses of multinomial logistic regression on identity statuses.

Identity statuses	Reference group	Online prosocial behavior	Offline prosocial behavior	Internet usage time	Sex; (0 = men; 1 = women)	Family income	Mother’s education (0 = secondary education; 1 = higher education)	Father’s education (0 = secondary school; 1 = higher school)	Region (0 = urban areas; 1 = rural areas)
		OR [95% CI]	OR [95% CI]	OR [95% CI]	OR [95% CI]	OR [95% CI]	OR [95% CI]	OR [95% CI]	OR [95% CI]
Searching moratorium	Achievement	1.60 ^**^ [1.18, 2.17]	0.76 [0.41, 1.40]	1.08 [0.92, 1.26]	1.12 [0.69, 1.79]	0.87 [0.75, 1.01]	1.01 [0.52, 1.97]	1.17 [0.61, 2.24]	1.20 [0.67, 2.16]
	Undifferentiated	2.07 ^***^ [1.59, 2.68]	2.82^***^ [1.70, 4.71]	1.00 [0.88, 1.13]	1.21 [0.80, 1.83]	0.94 [0.82, 1.07]	1.10 [0.63, 1.91]	1.44 [0.84, 2.46]	1.07 [0.66, 1.74]
	Troubled diffusion	2.83 ^***^ [2.13, 3.76]	1.72 [0.98, 3.01]	0.97 [0.85, 1.12]	0.75 [0.47, 1.19]	1.02 [0.88, 1.19]	1.05 [0.58, 1.89]	1.75 [0.99, 3.08]	1.21 [0.71, 2.06]
	Carefree diffusion	2.95 ^***^ [2.18, 4.00]	4.10^***^ [2.19, 7.69]	0.93 [0.80, 1.07]	1.38 [0.85, 2.23]	1.06 [0.90, 1.25]	1.50 [0.79, 2.84]	1.35 [0.73, 2.49]	1.21 [0.69, 2.10]
Achievement	Undifferentiated	1.30^*^ [1.00, 1.67]	3.74^***^ [2.14, 6.55]	0.92 [0.80, 1.07]	1.09 [0.70, 1.69]	1.08 [0.95, 1.23]	1.08 [0.59, 1.98]	1.23 [0.68, 2.21]	0.89 [0.52, 1.54]
	Troubled diffusion	1.77^***^ [1.35, 2.33]	2.28^**^ [1.26, 4.12]	0.90 [0.77, 1.06]	0.67 [0.42, 1.09]	1.18^*^ [1.01, 1.37]	1.03 [0.55, 1.95]	1.49 [0.81, 2.74]	1.01 [0.56, 1.80]
	Carefree diffusion	1.85^***^ [1.38, 2.49]	5.43^***^ [2.80, 10.54]	0.86 [0.73, 1.01]	1.24 [0.74, 2.05]	1.22^*^ [1.03, 1.43]	1.48 [0.75, 2.91]	1.15 [0.60, 2.22]	1.01 [0.55, 1.84]
Undifferentiated	Troubled diffusion	1.37^**^ [1.12, 1.68]	0.61^*^ [0.39, 0.94]	0.98 [0.87, 1.10]	0.62^*^ [0.42, 0.92]	1.09 [0.96, 1.24]	0.96 [0.60, 1.53]	1.21 [0.78, 1.90]	1.13 [0.72, 1.76]
	Carefree diffusion	1.43^**^ [1.14, 1.79]	1.45 [0.88, 2.40]	0.93 [0.82, 1.05]	1.14 [0.76, 1.71]	1.13 [0.98, 1.30]	1.37 [0.83, 2.26]	0.94 [0.57, 1.53]	1.13 [0.72, 1.77]
Troubled diffusion	Carefree diffusion	1.04 [0.82, 1.33]	2.38^**^ [1.40, 4.06]	0.95 [0.84, 1.08]	1.84^**^ [1.17, 2.88]	1.04 [0.89, 1.21]	1.43 [0.83, 2.46]	0.77 [0.46, 1.30]	1.00 [0.60, 1.67]

OR, odds ratio; 95% CI, 95% confidence interval.

^*^p < 0.05, ^**^p < 0.01, and ^***^p < 0.001.

## 4. Summary and discussion

In today’s online environment where we can easily engage with others, online prosocial behavior may play an important role in identity development, the most crucial developmental task in adolescence (Erikson, 1968; Branje et al., 2021), as offline prosocial behavior. We aimed to examine the associations of online and offline prosocial behavior with identity development among early and middle adolescents, using variable- and person-centered approaches.

In the variable-centered approach, consistent with Hypothesis 1a, both online and offline prosocial behaviors were positively related to commitments and proactive explorations among early and middle adolescents. These findings indicate that prosocial behaviors in online contexts, in addition to offline contexts as found in previous studies, make new contributions to identity development in adolescence. Co-construction theory assumes that there is a psychological continuum between adolescents’ offline and online activities, and that adolescents address developmental issues through interactions with others in online environments as in offline environments (Subrahmanyam et al., 2006, 2008). Based on this theory, as offline prosocial behavior involves a sense of responsibility, role, and agency in society (Yates and Youniss, 1996), online prosocial behavior may give adolescents an opportunity to experience these feelings. Hence, online prosocial behavior may encourage adolescents to consider and choose their future lives in society as identity options. In addition, contrary to Hypothesis 1b, there was no association between online prosocial behavior and ruminative exploration among early adolescents. Early adolescents cannot use the internet well because they use it impulsively and sensitively (Liu et al., 2022), as online prosocial behavior is associated with online antisocial behavior (Erreygers et al., 2018). This may be attributed to an excessive inclination toward maintaining connections with online communities (Liu et al., 2022). Such connections with online communities may lead some adolescents into ruminative exploration and others out of ruminative exploration. Therefore, associations between online prosocial behavior and maladaptive identity dimensions may not have been found in early adolescence.

In the person-centered approach, early and middle adolescents with higher levels of online prosocial behavior were more likely to show *searching moratorium* than other statuses, including *achievement*, whereas adolescents with higher levels of offline prosocial behavior were more likely to show *achievement* than immature and intermediate identity statuses. These results generally supported Hypotheses 2a–c, except for the absence of age-related differences in these relationships. Prior research has indicated that interacting with others in online environments might be a beneficial activity for adolescents who want to explore their own identity, because it allows them to freely explore their various values without being constrained by social norms in the real world (Jordán-Conde et al., 2014; Moreno and Uhls, 2019; Armstrong-Carter and Telzer, 2021). Therefore, our results suggest that early and middle adolescents who engage in online prosocial behavior actively explore their sense of belonging and social roles in online environments and, as a result, might show *searching moratorium*. Moreover, adolescents with high levels of offline prosocial behavior are committed to their identity-related choices by gaining a sense of agency in the real society even in today’s digital age, and thus they might show *achievement*; this accords with previous studies (Pancer et al., 2007; Hardy et al., 2011).

Overall, the results highlight that the importance of both online and offline prosocial behaviors in identity development among contemporary adolescents. In particular, consistent with both variable- and person-centered approaches, online prosocial behavior serves as a new resource for developing identity. Moreover, the results suggest that engaging in online prosocial behavior leads to identity status that is not satisfied with current commitments and continues to explore further, and that engaging in offline prosocial behaviors was necessary to become more mature identity status. In summary, this study adds significant knowledge regarding the impact of online and offline prosocial behaviors on adaptive identity development in adolescence, a topic that has received relatively little attention in previous research.

### 4.1. Practical implications

This study provides two practical implications for adolescents’ identity development in today’s digitalized society. First, it may be useful to encourage adolescents to engage in prosocial behavior in online environments as the first trigger to facilitate identity development during adolescence. Our results revealed that adaptive identity dimensions were facilitated by online prosocial behaviors and that adolescents engaging in online prosocial behaviors were more likely to show *searching moratorium* status in the process of maturing identity. Adolescents have opportunities to easily engage in online prosocial behaviors using electronic devices, because online environments have fewer temporal and social constraints than in-person environments (Armstrong-Carter and Telzer, 2021). Through such online technology, adolescents in a marginalized society may also be able to participate in society by communicating with diverse others (Manago, 2015). In addition, considering intervention techniques to improve mental health through online text message exchanges (Nitzburg and Farber, 2019); it may be important to provide opportunities and methods to promote identity development through prosocial behavior using online text messages. Therefore, it is crucial for adults around adolescents to convey to them the significance of online media literacy, including being socially responsible and supportive when interacting with others in online spaces, to explore their identity.

Second, encouraging adolescents to engage in prosocial behavior in offline environments may be helpful for further promoting their identity development. Our results showed that adolescents who actively engaged in offline prosocial behavior were more likely to be *achievement* in the matured identity status. Although adolescents are more likely to engage in online prosocial behavior than offline prosocial behavior in this digital age owing to reduced social and temporal constraints, offline prosocial behavior is still important for more adaptive development in adolescence (Armstrong-Carter and Telzer, 2021). Adolescents participating in volunteering programs in the community have gained agency, which is a source of identity (Webb et al., 2017). Thus, for adolescents who are exploring their identity through online prosocial behavior, as the next step, it is necessary for adults to create opportunities for prosocial behavior in offline environments to help adolescents develop more mature identity in the real society.

### 4.2. Limitations and future research directions

The current study has several strengths compared to previous studies. This study presents the first empirical findings on the role of online prosocial behavior in identity development during adolescence, provides a comprehensive understanding of these relationships using both variable- and person-centered approaches, and examines these developmental relationships in two age groups: early and middle adolescence. Despite these strengths, this study had some limitations. First, the online and offline prosocial behavior scales in this study did not measure behaviors themselves, and these scales captured different behavioral dimensions of prosocial behaviors (i.e., behavioral dispositions and behavioral tendencies). Future studies need to assess actual online and offline prosocial behaviors (e.g., examining the number of messages about prosocial behavior sent via SNS and volunteers participating in in-person environments). In addition, future research should also create scale items for prosocial behaviors that are applicable both offline and online contexts to align the behavioral dimensions in the scales. Second, this study used cross-sectional data. Future research should use longitudinal data to confirm whether online and offline prosocial behaviors predict identity development. Third, this study compared early and middle adolescents to examine developmental associations and found no differences between the two groups. As social desirability bias can influence the measurement of online prosocial behavior in adolescents (Lysenstøen et al., 2021), the variance of responses between age groups may be smaller. Future studies need to examine these associations controlling for social desirability. Fourth, it is important to consider to whom prosocial behavior is directed in future research. Given that online prosocial behavior is more often directed toward anonymous others than is offline prosocial behavior (Armstrong-Carter and Telzer, 2021), it is necessary to investigate whether the association between identity development and prosocial behavior depends on the recipients of prosocial behavior. Finally, we examined the associations of online and offline prosocial behavior with the processes of identity development (i.e., exploration and commitment), but to take an integrated view of identity development (Galliher et al., 2017), future studies should examine the relationships with identity content (e.g., positive and negative valences of identity; Hihara et al., 2022), an important aspect of identity development.

## 5. Conclusion

The current study presents novel and significant findings regarding the relationship between identity development and online and offline prosocial behavior in adolescence. It should be noted that the online and offline prosocial behaviors scales used in this study do not necessarily capture the behaviors themselves. Our results obtained from both the variable- and person-centered approaches revealed that online prosocial behavior could be a new resource for identity development in adolescence. Moreover, adolescents with high levels of online prosocial behavior were more likely to show identity status in the maturing process, while adolescents with high levels of offline prosocial behavior were more likely to show matured identity status. These results provide practical implications regarding how adults should create opportunities for online and offline prosocial behaviors according to the maturation stage of adolescents’ identity development. We hope that our findings will provide new directions for future research on identity development and prosocial behavior among adolescents in the digital age.

## Data availability statement

The raw data supporting the conclusions of this article will be made available by the authors, without undue reservation.

## Ethics statement

The studies involving human participants were reviewed and approved by Ethical Review Board in Hiroshima University. Written informed consent to participate in this study was provided by the participants’ legal guardian/next of kin.

## Author contributions

YI developed the research design and collected data with SH. YI performed statistical analyses and wrote the first draft of the manuscript. SH, KI, GY, MH, and KS critically revised the manuscript. YI, SH, KI, GY, MH, and KS contributed to data interpretation, reviewed this manuscript, and approved the submission. All authors contributed to the article and approved the submitted version.

## Funding

This study was supported by a Japan Society for the Promotion of Science Grant-in-Aid for JSPS Fellows (Grant number 20J11894) and Research Activity Start-up (Grant number 22K20310).

## Conflict of interest

The authors declare that the research was conducted in the absence of any commercial or financial relationships that could be construed as a potential conflict of interest.

## Publisher’s note

All claims expressed in this article are solely those of the authors and do not necessarily represent those of their affiliated organizations, or those of the publisher, the editors and the reviewers. Any product that may be evaluated in this article, or claim that may be made by its manufacturer, is not guaranteed or endorsed by the publisher.

## Footnotes

^1^The individuals in middle adolescence were calculated by removing five participants who answered that they were 17-years-old or older. Given that first grade high school students in Japan are generally 15- or 16-years-old, those aged 17 and above were considered to follow rare educational paths in Japan such as repeating a year and to be outliers. Therefore, they were omitted from this study.
