# Supplementary material for: Identity development and online and offline prosocial behaviors among early and middle adolescents
Source: Front Psychol. 2023 May 25;14:1148347. doi: 10.3389/fpsyg.2023.1148347 (PMC10248000; doi:10.3389/fpsyg.2023.1148347)
Supplement: Supplementary file 1 [file Table_1.docx]

Supplementary Materials

# Measurement Invariance Tests

To test measurement invariance, confirmatory factor analyses were conducted according to the processes described in the Strategy of Analyses section of the manuscript. It is suggested to use parceling techniques when the scale contains more than five items for each construct (Bagozzi and Heatherton, 1994). Parcels of items for each construct in identity development and online prosocial behavior were constructed and used as indicators of the latent variables. Specifically, in identity development, two parcels and one parcel included two items and one item, respectively. In online prosocial behavior, two parcels consisted of three items and the other two parcels consisted of two items. As shown in Table S1, the results of the measurement invariance tests revealed that scalar invariance was present for each construct examined in the study.

Bagozzi, R. P., and Heatherton, T. F. (1994). A general approach to representing multifaceted personality constructs: Application to state self‐esteem. *Struct. Equ. Modeling* 1, 35–67. doi: 10.1080/10705519409539961.

Table S1. Tests of measurement invariance across age groups.

|  |  | Model fit indices | | | | |  | Model comparisons | | | | | |
| --- | --- | --- | --- | --- | --- | --- | --- | --- | --- | --- | --- | --- | --- |
|  |  | χ 2 SB | *df* | *p*-values | CFI | RMSEA [90% CI] |  | Pairs | Δχ 2 SB | Δ*df* | *p*-values | ΔCFI | ΔRMSEA |
| Identity development | |  |  |  |  |  |  |  |  |  |  |  |  |
|  | M1: Configural invariant model | 518.960 | 160 | < 0.001 | 0.964 | 0.061 [0.055, 0.067] |  |  |  |  |  |  |  |
|  | M2: Metric invariant model | 530.534 | 170 | < 0.001 | 0.964 | 0.059 [0.054, 0.065] |  | M2-M1 | 4.738 | 10 | 0.908 | 0.000 | -0.002 |
|  | M3: Scalar invariant model | 587.475 | 185 | < 0.001 | 0.960 | 0.060 [0.055, 0.066] |  | M3-M2 | 59.570 | 15 | < 0.001 | -0.002 | 0.001 |
| Online prosocial behavior | |  |  |  |  |  |  |  |  |  |  |  |  |
|  | M1: Configural invariant model | 15.093 | 4 | < 0.001 | 0.995 | 0.068 [0.034, 0.106] |  |  |  |  |  |  |  |
|  | M2: Metric invariant model | 20.293 | 7 | 0.005 | 0.994 | 0.056 [0.029, 0.086] |  | M2-M1 | 2.547 | 3 | 0.467 | -0.001 | -0.012 |
|  | M3: Scalar invariant model | 38.429 | 11 | < 0.001 | 0.987 | 0.064 [0.043, 0.087] |  | M3-M2 | 18.681 | 4 | 0.001 | -0.007 | 0.008 |
| Offline prosocial behavior | |  |  |  |  |  |  |  |  |  |  |  |  |
|  | M1: Configural invariant model | 24.876 | 10 | < 0.001 | 0.979 | 0.050 [0.025, 0.075] |  |  |  |  |  |  |  |
|  | M2: Metric invariant model | 32.438 | 14 | < 0.001 | 0.974 | 0.047 [0.026, 0.068] |  | M2-M1 | 7.202 | 4 | 0.126 | -0.005 | -0.003 |
|  | M3: Scalar invariant model | 44.404 | 19 | < 0.001 | 0.964 | 0.047 [0.029, 0.065] |  | M3-M2 | 12.335 | 5 | 0.030 | -0.010 | 0.000 |

*Note*. M = Model; χ 2 SB = Satorra-Bentler chi-square; *df* = degrees of freedom; CFI = comparative fit index; RMSEA = root mean square error of approximation; CI = confidence interval; Δ = change in parameter.

| Variables | Sex | | | | Father's educational background | | | | Mother's educational level | | | | Region | | | |
| --- | --- | --- | --- | --- | --- | --- | --- | --- | --- | --- | --- | --- | --- | --- | --- | --- |
|  | Men | Women |  |  | Secondary  education | Higher   education |  |  | Secondary   education | Higher   education |  |  | Urban areas | Rural areas |  |  |
|  | *M* (*SD*) | *M* (*SD*) | *t*-values | Cohen’s *d* | *M* (*SD*) | *M* (*SD*) | *t*-values | Cohen’s *d* | *M* (*SD*) | *M* (*SD*) | *t*-values | Cohen’s *d* | *M* (*SD*) | *M* (*SD*) | *t*-values | Cohen’s *d* |
| Commitment  making | 2.96 (1.05) | 3.09 (1.01) | *t* (1200) = 2.16^*^ | 0.12 | 2.85 (1.02) | 3.10 (1.02) | *t* (1191) = 3.79^***^ | 0.24 | 2.89 (1.04) | 3.07 (1.02) | *t* (1200) = 2.77^**^ | 0.18 | 3.06 (1.02) | 2.95 (1.04) | *t* (1200) = 1.61 | 0.11 |
| Identification with commitment | 3.07 (0.94) | 3.12 (0.86) | *t* (1200) = 1.09 | 0.06 | 2.96 (0.92) | 3.15 (0.89) | *t* (1191) = 3.36^**^ | 0.21 | 2.96 (0.94) | 3.14 (0.88) | *t* (1200) = 3.11^**^ | 0.21 | 3.12 (0.89) | 3.03 (0.94) | *t* (1200) = 1.56 | 0.10 |
| Exploration  in breadth | 3.16 (0.92) | 3.32 (0.85) | *t* (1200) = 3.09^**^ | 0.18 | 3.08 (0.87) | 3.31 (0.86) | *t* (1191) = 4.24^***^ | 0.27 | 3.09 (0.93) | 3.29 (0.87) | *t* (1200) = 3.56^***^ | 0.24 | 3.27 (0.87) | 3.17 (0.92) | *t* (1200) = 1.61 | 0.11 |
| Exploration  in depth | 2.95 (0.88) | 3.14 (0.86) | *t* (1200) =  3.86^**^ | 0.22 | 2.92 (0.89) | 3.10 (0.86) | *t* (1191) = 3.28^**^ | 0.21 | 2.93 (0.89) | 3.08 (0.86) | *t* (1200) =  2.59^*^ | 0.17 | 3.06 (0.87) | 3.00 (0.89) | *t* (1200) = 1.00 | 0.07 |
| Ruminative  exploration | 3.10 (0.78) | 3.21 (0.81) | *t* (1200) =  2.35^*^ | 0.14 | 3.12 (0.80) | 3.17 (0.80) | *t* (1191) = 0.92 | 0.06 | 3.09 (0.83) | 3.17 (0.79) | *t* (1200) =  1.53 | 0.10 | 3.15 (0.80) | 3.15 (0.80) | *t* (1200) = 0.06 | 0.00 |
| Online prosocial behavior | 2.47 (0.95) | 2.79 (1.02) | *t* (1200) =  5.68^***^ | 0.33 | 2.53 (0.95) | 2.67 (1.02) | *t* (1191) = 2.20^*^ | 0.14 | 2.50 (0.97) | 2.67 (1.00) | *t* (1200) =  2.64^**^ | 0.18 | 2.67 (1.00) | 2.53 (0.99) | *t* (1200) =2.12^*^ | 0.14 |
| Offline prosocial behavior | 1.03  (0.44) | 1.10 (0.45) | *t* (1200) = 2.79^**^ | 0.16 | 1.04 (0.44) | 1.07 (0.45) | *t* (1191) = 1.09 | 0.07 | 1.06 (0.46) | 1.07 (0.45) | *t* (1200) = 0.19 | 0.01 | 1.08 (0.45) | 1.03 (0.45) | *t* (1200) = 1.75 | 0.11 |

Table S2. T-test results of sex, parents’ educational level and region on the study variables.

*Note*. *M* = mean; S*D* = standard deviation.

^*^ *p* < 0.05, ^**^ *p* < 0.01, ^***^ *p* < 0.001
